# Fibrinogen may aid in the early differentiation between amniotic fluid embolism and postpartum haemorrhage: a retrospective chart review

**DOI:** 10.1038/s41598-021-87685-y

**Published:** 2021-04-16

**Authors:** Shigetaka Matsunaga, Hiroko Masuko, Yasushi Takai, Naohiro Kanayama, Hiroyuki Seki

**Affiliations:** 1 Center for Maternal, Fetal and Neonatal Medicine, Saitama Medical Center, Saitama Medical University, Saitama, 350-8550 Japan; 2 Department of Obstetrics and Gynecology, Hamamatsu University School of Medicine, Shizuoka, 431-3192 Japan

**Keywords:** Health care, Medical research

## Abstract

This study aimed to determine whether blood loss and fibrinogen can differentiate amniotic fluid embolism (AFE) from postpartum haemorrhage (PPH). This retrospective case–control study included nine patients with clinical AFE (“AFE group”) and 78 patients with PPH managed at our tertiary care perinatal centre between January 2014 and March 2016. Patients meeting the Japanese diagnostic criteria for AFE were stratified into cardiopulmonary collapse-type AFE and disseminated intravascular coagulation (DIC)-type AFE groups. The relationship between blood loss and fibrinogen at onset was examined to compare DIC severity. Vital signs at onset were not significantly different. The AFE group had significantly less blood loss at onset (1506 mL vs 1843 mL, P = 0.0163), significantly more blood loss 2 h post-onset (3304 mL vs 1996 mL, P < 0.0001) and more severe coagulopathy and fibrinolysis. The blood loss/fibrinogen (B/F) ratio at onset was significantly higher in the DIC-type AFE group (23.15 ± 8.07 vs 6.28 ± 3.35 mL dL/mg, P < 0.0001). AFE was complicated by catastrophic DIC irrespective of blood loss at onset. Fibrinogen exhibited the strongest correlation among test findings at onset. The B/F ratio may help differentiate PPH from DIC-type AFE and diagnose clinical AFE, facilitating optimal replacement of coagulation factors during the early stages.

## Introduction

Amniotic fluid embolism (AFE) is a rare, severe maternal complication with an incidence of 0.001–0.013%^1,2^, and the mortality rate is very high (37–80%)^3,4^. AFE reportedly causes 24.3% of all maternal deaths in Japan^5^.

AFE likely results from amniotic/foetal component-triggered physical embolism and an anaphylactoid reaction^3^. Definitive diagnosis of AFE comprises conventional autopsy findings of amniotic fluid/foetal components in maternal lung and uterine tissue, and interstitial oedema with inflammatory cell infiltration related to anaphylactic reactions^6,7^. Because autopsy results take time to produce definitive diagnoses, timely practical diagnostic criteria are needed to improve maternal life expectancy.

Recent analyses by Japan’s Maternal Death Exploratory Committee and the Amniotic Fluid Embolism Registry have raised the “concern that AFE is implicated in massive obstetric haemorrhage”. Disseminated intervascular coagulation (DIC) [uterine]-type AFE causes critical obstetric haemorrhage. Reports on this subject are increasing^5^. The perinatal committee of the Japanese Society of Obstetrics and Gynaecology described clinical features of uterine-type AFE to clarify the concept^8^.

Diagnostic criteria for clinical AFE were proposed based on the clinical presentation of cardiopulmonary collapse or DIC triggered by amniotic/foetal component inflow into maternal blood (Supplementary Table S1)^9,10^. However, there are problems with the current AFE diagnostic criteria. Although “severe bleeding of unknown origin within 2 h of delivery” and “DIC” are important diagnostic criteria, it is difficult to differentiate AFE and early-stage postpartum haemorrhage (PPH) unrelated to AFE (“non-AFE PPH”) without symptoms of cardiopulmonary collapse. DIC causes secondary atonic bleeding due to the relaxation of uterine smooth muscle. Excessive blood product resources and personnel may be utilised when atonic bleeding secondary to DIC and DIC secondary to atonic bleeding are conflated, and a diagnosis of DIC (uterine)-type AFE is reached. In non-AFE PPH, the amount of coagulation factors needed generally correlates with the amount of bleeding, whereas in DIC (uterine)-type AFE, severe coagulopathy should be anticipated from the early stages after its onset. Prompt replacement of much larger amounts of coagulation factors, specifically fibrinogen concentrates and cryoprecipitates, may be necessary.

DIC complicates early-stage classic AFE and DIC-type AFE in 50–80% of cases^4,5,11^. DIC-type AFE is characterised by consumption coagulopathy with a clear decrease in coagulation factors before blood loss increases^12,13^. Because bleeding triggered by severe DIC worsens quickly, coagulation factor laboratory values can resemble those of DIC after non-AFE PPH, depending on the length of time since onset. Therefore, it may be impossible to recognise the characteristic findings of AFE.

Our objective was to provide a comparative evaluation of haematological findings and blood loss in early-stage AFE and non-AFE PPH to clarify the features of early-stage AFE. We examined fibrinogen levels^14^ to assess the severity of DIC and whether it could be used to differentiate between AFE and non-AFE PPH.

## Methods

This study was approved by the ethics committee of Saitama Medical Center/Saitama Medical University (approval number: 1671). The requirement for informed consent was waived because this was a retrospective medical chart review. We compared nine cases of clinical AFE (“AFE group”) and 78 cases of PPH excluding clinical AFE (“non-AFE PPH group”) from the 2385 patients that our department managed for delivery and postpartum transport between January 2014 and March 2016 (Supplementary Fig. S1). This study adhered to the principles of the Declaration of Helsinki. We declare that this study did not receive any external sources of funding.

The AFE group comprised patients who met the Japan consensus criteria for the diagnosis of AFE^10^, which are based on American and British diagnostic criteria. The non-AFE PPH group, serving as the control, comprised patients who experienced massive blood loss of 1500 mL or more within 2 h after delivery, based on the diagnostic criteria for clinical amniotic fluid embolism. In addition, the clinical features of DIC-type AFE were as follows: (1) non-coagulable bleeding accompanied by marked coagulopathy immediately after placental delivery; (2) soft uterine muscle, with the uterine fundal height reaching two or more fingers above the navel; and (3) uterine atony that does not respond to uterine contraction agents^10,15^. These patients subsequently underwent haematological testing, and any patients with diseases (i.e., thrombocytopaenia, sepsis, systemic inflammatory response syndrome) that could have caused coagulation factor disorders or placental abruption, leading to a consumption coagulopathy similar to AFE, were excluded.

The “(time of) onset” was regarded as the point in time when diagnostic, criteria-based symptoms of clinical AFE were observed in the AFE group and the point in time when blood loss of 1500 mL or more was detected in the non-AFE PPH group. The vital signs at onset (systolic blood pressure, heart rate, shock index), blood loss at onset (equivalent to blood loss at the time of blood testing), blood loss at 2 h after onset, haematological findings, and obstetric DIC scores were examined.

Measurements were taken by several expert nurses trained in the management of bleeding during labour and surgery, similar to our previous studies^12,16^. To determine the total amount of blood loss, real-time measurements of the amount of blood extracted into the aspirator and the weight of blood that infiltrated into the absorption pads and gauze were measured every 20 min. The weight of blood that infiltrated into the surgical drapes was also measured after each procedure.

The blood loss (B) was divided by the fibrinogen levels (F) at onset to calculate the B/F ratio (mL dL/mg) and to compare the extent of consumption coagulopathy at onset.

Statistical analysis was performed using JMP v13.0 (SAS Institute, Cary, NC, USA). The Kruskal–Wallis and chi-square tests were used to compare the two groups, with the significance level set to a p-value less than 0.05.

## Results

Within the study period, 2385 patients were managed for delivery and postpartum at our department, of whom the records of 128 patients indicated massive blood loss of 1500 mL or more. After applying the exclusion criteria, 87 patients’ cases were used in the analysis. None of these patients received anticoagulant or antiplatelet therapy.

The AFE group included nine patients, with three cases of cardiopulmonary collapse-type AFE (patients 1–3) and six cases of DIC-type (uterine-type) AFE (patients 4–9). The patients’ backgrounds are shown in Table 1. Patients 1, 2, and 3 had cardiopulmonary collapse symptoms and impaired consciousness. The conditions of patients 1 and 2 resulted in maternal deaths, and patient 3 had neurological sequelae. Autopsy consent was obtained for one of the two fatalities, and the cause of death was verified as AFE.

**Table 1:** Clinical characteristics of the patients with AFE.

No.	Patient	Maternal outcome	New-born outcome	Risk factor	Onset	Presenting symptoms	STN	Zn-CP1
							< 45.0 (U/mL)	< 1.6 (pmol/mL)
1	36 y.o. multipara	Death	IUFD	–	During labour	NRFS, consciousness disorder	46	0.18
2	43 y.o. multipara	Death	CP	–	During labour	Dyspnoea, consciousness disorder	500	9.3
3	32 y.o. primipara	Neurologic sequelae	Neonatal asphyxia	–	During labour	Dyspnoea, consciousness disorder	440	< 1.6
4	39 y.o. multipara	Alive	Alive	CS	During CS	Non-coagulable massive bleeding^a^	40	< 1.6
5	41 y.o primipara	Alive	Alive	CS	During CS	Non-coagulable massive bleeding^a^	< 1.6	17
6	19 y.o. primipara	Alive	Alive	Amniotic reduction	After delivery	Non-coagulable massive bleeding^a^	< 1.6	21
7	35 y.o. primipara	Alive	Alive	–	After delivery	Non-coagulable massive bleeding^a^	–	–
8	38 y.o. multipara	Alive	Alive	Foetal therapy, CS	Just after CS	Non-coagulable massive bleeding^a^	31	0.7
9	32 y.o primipara	Alive	Alive	CS	Just after CS	Non-coagulable massive bleeding^a^	< 5.0	< 1.6

*AFE* amniotic fluid embolism, *IUFD* intrauterine foetal death, *CP* cerebral palsy, *CS* caesarean section, *NRFS* non-reassuring foetal status, *STN* sialyl Tn, *Zn-CP1* zinc coproporphyrin.

^a^See text for details.

Patients 4–9 all survived after massive blood losses subsequent to delivery (during Caesarean section) or 2 h after delivery (post-Caesarean section). All nine patients fulfilled every clinical feature of DIC-type AFE, including non-coagulable bleeding immediately after placental delivery. At the time of onset, the main symptom was non-coagulable bleeding accompanied by marked coagulopathy immediately after placental delivery and uterine atony that did not respond to uterine contraction agents.

The 78 pregnancies in the non-AFE PPH group were complicated by twin pregnancies, uterine atony, low-lying placenta/placenta previa/placenta accreta, and birth canal lacerations. Supplementary Table S2 shows the patients’ backgrounds for the groups. There was no significant difference in maternal age or the proportion of multiparous patients between the two groups. The use of labour induction agents was significantly higher in the AFE group (4/9 (44.4%) vs 6/78 (7.7%); P = 0.001), and the rate of Caesarean sections was significantly lower in the AFE group (4/9 (44.4%) vs 66/78 (84.6%); P = 0.004).

There were no significant differences in vital signs at onset between the two groups. The AFE group had significantly lower amounts of blood loss at onset (AFE group: median 1506 mL vs non-AFE PPH group: median 1843 mL, P = 0.0163) but had significantly higher amounts of blood loss at 2 h after onset (median 3304 mL vs median 1996 mL, P < 0.0001) (Supplementary Table S3).

There were no significant differences between the two groups in haemoglobin levels, but the platelet counts were lower, and the blood coagulative/fibrinolytic function values were higher in the AFE group. The obstetrical DIC score, which indicates the severity of DIC, was also high in the AFE group (Table 2).

**Table 2:** Comparison of haemostatic parameters at onset for each group.

	AFE group (n = 9) Median (range)	Mean ± SD	Non-AFE PPH group (n = 78) Median (range)	Mean ± SD	P-value
Hb (g/dL)	8.1 (3.1–13.7)	7.9 (± 2.9)	9.0 (5.0–12.7)	9.0 (± 1.6)	0.0935
PLT (10^4^/μL)	8.5 (7–18.3)	11.0 (± 4.3)	17.8 (6.3–34.1)	18.0 (± 5.5)	0.0004*
aPTT (sec)	78.2 (33–180)	86.2 (± 46.4)	30.6 (21.8–88.4)	32.0 (± 7.7)	< 0.0001*
PT (%)	16 (10–53)	27.3 (± 17.2)	87 (27.9–100)	84.6 (± 12.7)	< 0.00001*
PT-INR	4.94 (1.44–5.2)	3.64 (± 1.80)	1.09 (0.87–1.73)	1.12 (± 0.13)	< 0.0001*
Fibrinogen (mg/dL)	70 (70–132)	76.9 (± 20.7)	327 (120–610)	336 (± 99.6)	< 0.0001*
FDP (μg/mL)	80 (80–80)	80.0 (± 0)	10.3 (5.36–80)	20.0 (± 19.9)	< 0.0001*
Obstetrical DIC Score	11 (5–25)	13.7 (± 7.4)	3 (1–23)	3.6 (± 3.0)	< 0.0001*

*Hb* haemoglobin concentration (normal range 11.6–14.8 g/dL), *PLT* platelet count (normal range 15.8–34.8 10^4^/μL), *aPTT* activated partial thromboplastin time (normal range 24–39 s), *PT (%)* prothrombin time activity percentage (normal range 80–120%), *PT-INR* International Normalized Ratio of Prothrombin Time (normal range 0.90–1.10), *FDP* fibrin degradation products (normal range 0.00–4.99 μg/mL), *DIC* disseminated intravascular coagulation, *AFE* amniotic fluid embolism, *SD* standard deviation, *PPH* postpartum haemorrhage.

* denotes significant difference.

Univariate analysis of vital signs and coagulative/fibrinolytic function tests showed a higher area under the curve (AUC) in the group diagnosed with clinical AFE (Table 3). The greatest AUC and strongest correlation were observed in the fibrinogen levels (AUC 1.00).

**Table 3:** Univariate analysis with ranks of haemostatic parameters at onset indicating correlation with AFE.

	AUC	95% CI	Rank
Systolic blood pressure	0.8167	0.6397–0.9179	7
Heart rate	0.7292	0.3486–0.9313	9
Shock index	0.85	0.4275–0.9773	6
Fibrinogen	1		1
PT-INR	0.9917	0.8791–0.9995	2
aPTT	0.9333	0.6725–0.9896	4
FDP	0.9667	0.8764–0.9916	3
Hb	0.5833	0.2807–0.8340	10
PLT	0.8125	0.5488–0.9392	8
Obstetrical DIC score	0.9125	0.7003–0.9790	5

*PT-INR* prothrombin-international normalised ratio, *aPTT* activated partial thromboplastin time, *FDP* fibrinogen degradation products, *Hb* haemoglobin, *PLT* platelet count, *DIC* disseminated intravascular coagulation, *AUC* area under the receiver operating characteristic curve, *CI* confidence interval.

When the cut-off values were calculated for each coagulative function test value, with respect to AFE positivity and using a receiver operating characteristic (ROC) curve based on the Youden index (sensitivity + specificity − 1), those of the tests with top three AUC values were 132 mg/dL (fibrinogen), 1.44 (PT-INR), and 80.0 μg/dL (fibrinogen degradation products [FDP]). The predictive power (PP), defined as (true positive + true negative)/total, was evaluated using the optimum cut-off value selected. The PP values for fibrinogen, PT-INR, and FDP were 0.9673, 0.9767, and 0.9512, respectively (Supplementary Fig. S2).

**Figure 1:**
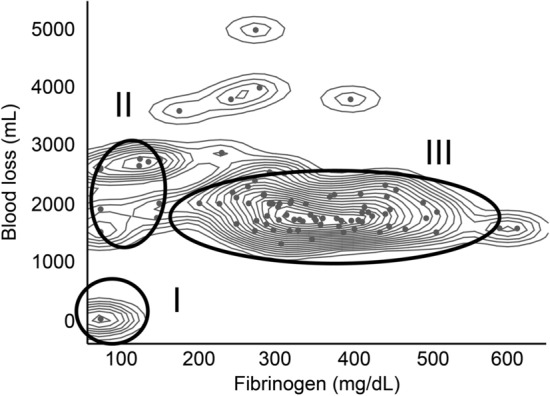
Non-parametric density estimation distribution of the ratio (B/F) of blood loss at onset and blood fibrinogen levels. Of all 87 cases shown, based on blood loss at onset and blood fibrinogen levels, the patients are divided as follows: group I: low-bleeding, low-fibrinogen group; group II: a heavy-bleeding, low-fibrinogen group; group III: a heavy-bleeding group that maintained stable fibrinogen levels. Group I included all three cases of systemic AFE (classic amniotic fluid embolism). Group II included six cases of disseminated intravascular coagulation-type (uterine-type) AFE. Seventy-three of the non-AFE post-partum haemorrhage cases (73/78 = 93.6%) belonged to group III, and the remaining five cases (5/78 = 6.4%) belonged to group II. The mean ± standard deviation B/F ratio was 23.15 ± 8.07 mL dL/mg for group II vs 6.28 ± 3.35 mL dL/mg for group III (P < 0.0001). The cut-off value from the receiver operating characteristic curve for groups II and III was 12.23, while the area under the curve was 0.9863, and the predictive power was 0.9404.

In a nonparametric density estimation of the 87 patients analysed, the patients were grouped based on the B/F ratio. The B/F ratio showed a broad grouping of patients with little blood loss and low fibrinogen levels (group I), those with blood loss equivalent to group III but with lower fibrinogen levels (group II), and those who maintained their fibrinogen levels (group III) (Fig. 1). The patients in group I matched the cardiopulmonary collapse-type AFE with a B/F ratio of 0.0143 ± 0.00 mL dL/mg. Group II matched the DIC (uterine)-type AFE, and most of the patients with PPH were included in group III (73/78 = 93.6%). However, group II also included five patients that were not diagnosed with clinical AFE. The B/F ratios for groups II and III, the patients of whom had similar blood losses at onset, were 23.15 ± 8.07 and 6.28 ± 3.35 mL dL/mg, respectively, a significant difference (P < 0.0001). According to the ROC analysis, the cut-off value for differentiating groups II and III was 12.23, the AUC was 0.9863, and the PP was 0.9404.

## Discussion

Compared to the PPH group, the AFE group had a significantly lower concentration of extrinsic coagulation factors, a representative example is fibrinogen. Fibrinogen exhibited the strongest correlation with AFE in our analysis, with a cut-off value at 132 mg/dL. Although the AFE group had significantly lower amounts of blood loss at onset than the PPH group, the extent of coagulopathy was severe, suggesting that the B/F ratio could be used to differentiate between these two diseases, both of which present with massive bleeding. Interestingly, despite similar amounts of bleeding being noted at onset, the fibrinogen level was significantly lower in patients with DIC-type AFE than those with non-AFE PPH. All patients in the AFE group exhibited DIC, and even patients that did not have bleeding developed severe consumption coagulopathy.

The nonparametric density estimation showed a group that exhibited a relatively small amount of bleeding, not accompanied by systemic symptoms, which were labelled “group II”, believed to consist of patients of the DIC (uterine)-type. However, only 6 out of 11 patients (54.5%) with extreme hyperfibrinogenaemia met the existing diagnostic criteria for clinical AFE. Conversely, the fact that five patients (45.5%) did not fulfil those diagnostic criteria suggests that the current diagnostic criteria for clinical AFE may not be adequate. In this disorder, which requires intensive treatment from its early stages, determining blood loss and quickly measuring fibrinogen could promote more accurate diagnosis and treatment.

We previously reported the usefulness of fibrinogen levels for predicting massive obstetrical haemorrhage^12^. Of the predictive factors for cases with massive bleeding, fibrinogen showed the strongest correlation, even more than the shock index or obstetrical DIC score. Fibrinogen is also reportedly useful as a criterion for assessing the severity of consumption coagulopathy^14^. The cut-off value for fibrinogen at which six or more units of RBC transfusion are needed is 155 mg/dL. In our analysis, fibrinogen exhibited the strongest correlation with AFE, consistent with previous reports, but the AUC, which shows the strength of the correlation, was higher, and the cut-off value was lower than in previous reports. The AFE group experienced more severe coagulopathy and differentiation according to their fibrinogen levels, a marker that appears to be more accurate than findings of massive bleeding or placental abruption, which was used in previous reports.

The mechanism by which AFE triggers DIC is based on the flow of amniotic fluid into the maternal blood. AFE occurs if there is a maternal anaphylactoid reaction to the amniotic fluid. C1-inhibitor plays a central role among the substances that suppress anaphylactoid reactions, and it has been suggested that anaphylactoid reactions occur when amniotic fluid enters the blood of patients with low C1-inhibitor levels^10,17^. When an anaphylactoid reaction occurs, factor XII and factor XI are activated, and plasminogen is activated in the fibrinolytic system. The kinin-kallikrein system is also activated, and bradykinin is produced by the plasmin-associated degradation of high-molecular-weight kininogen. Anaphylatoxins are activated as well as the complement system, which produces anaphylatoxins such as C3a and C5a. Because bradykinin acts as a smooth muscle relaxant^18,19^, it lowers blood pressure and worsens atonic bleeding. The entry of amniotic fluid tissue factors into the maternal bloodstream also activates the extrinsic system. Tissue thromboplastin is found in the amniotic fluid, and it is one possible cause of consumption coagulopathy^20^. When tissue thromboplastin enters the bloodstream, it results in excessive conversion of fibrinogen into fibrin, creating microthrombi. In the process, tissue thromboplastin forms a complex with factor VII and is consumed, thus lowering its concentration in the blood^21^. With this type of coagulopathy, the cause of DIC is different from the dilutional coagulopathy caused by infusion or transfusion or the loss of coagulation factors due to massive bleeding. Therefore, severe DIC can develop even if there is no massive bleeding caused by vascular collapse. The anaphylactoid reaction also includes a pathway wherein proteases derived from inflammatory cells, a representative example is granulocyte elastase, which directly breaks down fibrinogen without passing through fibrin. It is also possible that the anaphylactoid reaction contributes to the rapid decrease in fibrinogen.

Ultrasound and other examination techniques offer relatively rapid diagnosis at the time of onset of placental abruption. However, DIC (uterine)-type AFE with consumption coagulopathy secondary to the entry of tissue factors as described above only shows consumption coagulopathy and uterine relaxation, which are difficult to distinguish from atonic bleeding. Diagnosis in these cases is, therefore, difficult, and delays in diagnosis result in greater severity of the condition and a worse prognosis. Consumption coagulopathy involving these diseases is associated with an inflowing of tissue factors (DAIT). There is a more prominent decrease in fibrinogen associated with blood loss than with atonic bleeding, and the B/F value is higher in the former. In such cases, close attention to the risk of severe consumption coagulopathy and rapid application of haemostatic resuscitation may improve patient prognosis.

There were some limitations in this study that must be acknowledged. First, our facility is a high-volume centre for maternal emergencies, and the frequency of AFE, including DIC-type AFE, may have been higher than those reported in the literature. Second, in this study, the measurement limit values for the coagulation profiles included fibrinogen < 70 mg/dL, PT-INR > 5.2, and FDP 80 μg/dL. Because extremely abnormal coagulation/fibrinolysis occurs with AFE, increasing the measurement limits may enable discovering newer and more sensitive coagulative/fibrinolytic markers. Third, the small sample size, particularly for the AFE group, and the retrospective nature of the study may have biased our results; an analysis of cases in the future could shed more light on the differences at onset between AFE and PPH.

Critical challenges remain in this field. In this study, the onset of AFE was defined as the time at which diagnostic criteria for clinical AFE were observed, but currently, there are no serum markers for a clear diagnosis of AFE in the early stages. Therefore, there is a need for future research to develop serum markers enabling more accurate and rapid assessments of the results. Moreover, a nationwide analysis is needed to establish diagnostic criteria. As this would require specimens from the early stages of the disease, each region would need to have resources such as coagulation tests in place to collect specimens and record the blood loss at the time of onset, followed by the rapid performance of testing and treatment at higher-order medical care facilities. Research on the risk factors of AFE has been conducted in various nationwide studies^22–25^, resulting in different diagnostic criteria for each country^3,10,26–28^. Those criteria are somewhat variable. Although it is being sought, at this time there is no highly specific marker to aid in the diagnosis of AFE at onset^29–32^. However, most of the diagnostic criteria for AFE include “coagulopathy”. As coagulopathy develops, focusing on its features imparts the possibility to improve diagnostic accuracy leading to early intervention and improved prognosis.

In conclusion, clinical AFE includes severe DIC, even in patients with little (to no) blood loss. Blood fibrinogen levels showed the strongest correlation at onset, with a cut-off value of 132 mg/dL. The B/F ratio has the potential to provide a reference for differentiating between PPH and DIC (uterine)-type AFE, both of which present with massive haemorrhage. The mortality rate associated with AFE remains high, and improvement in its associated prognosis will require earlier diagnosis and therapeutic interventions. In light of our results, fibrinogen may improve early clinical diagnosis to improve the prognosis of AFE via multidisciplinary therapy in the early stages.

## Supplementary Information

Supplementary Information.

## Data Availability

No datasets were generated or analysed during the current study.
