# Supplementary material for: Fibrinogen may aid in the early differentiation between amniotic fluid embolism and postpartum haemorrhage: a retrospective chart review
Source: Sci Rep. 2021 Apr 16;11:8379. doi: 10.1038/s41598-021-87685-y (PMC8052446; doi:10.1038/s41598-021-87685-y)
Supplement: Supplementary file 1 — Supplementary Information. [file 41598_2021_87685_MOESM1_ESM.docx]

**Differentiation of clinical amniotic fluid embolism and postpartum haemorrhage to determine the onset of the amniotic fluid embolism**

Shigetaka Matsunaga^1*^, Hiroko Masuko^1^, Yasushi Takai^1^, Naohiro Kanayama^2^, Hiroyuki Seki^1^

^1^Center for Maternal, Fetal and Neonatal Medicine, Saitama Medical Center, Saitama Medical University, Saitama 350-8550, Japan.

^2^Department of Obstetrics and Gynecology, Hamamatsu University School of Medicine, Japan.

*Corresponding author (Phone: +81-49-228-3681; FAX: +81-49-226-1495. E-mail: [shige_m@saitama-med.ac.jp](mailto:shige_m@saitama-med.ac.jp))

**Supplementary Figures**

**
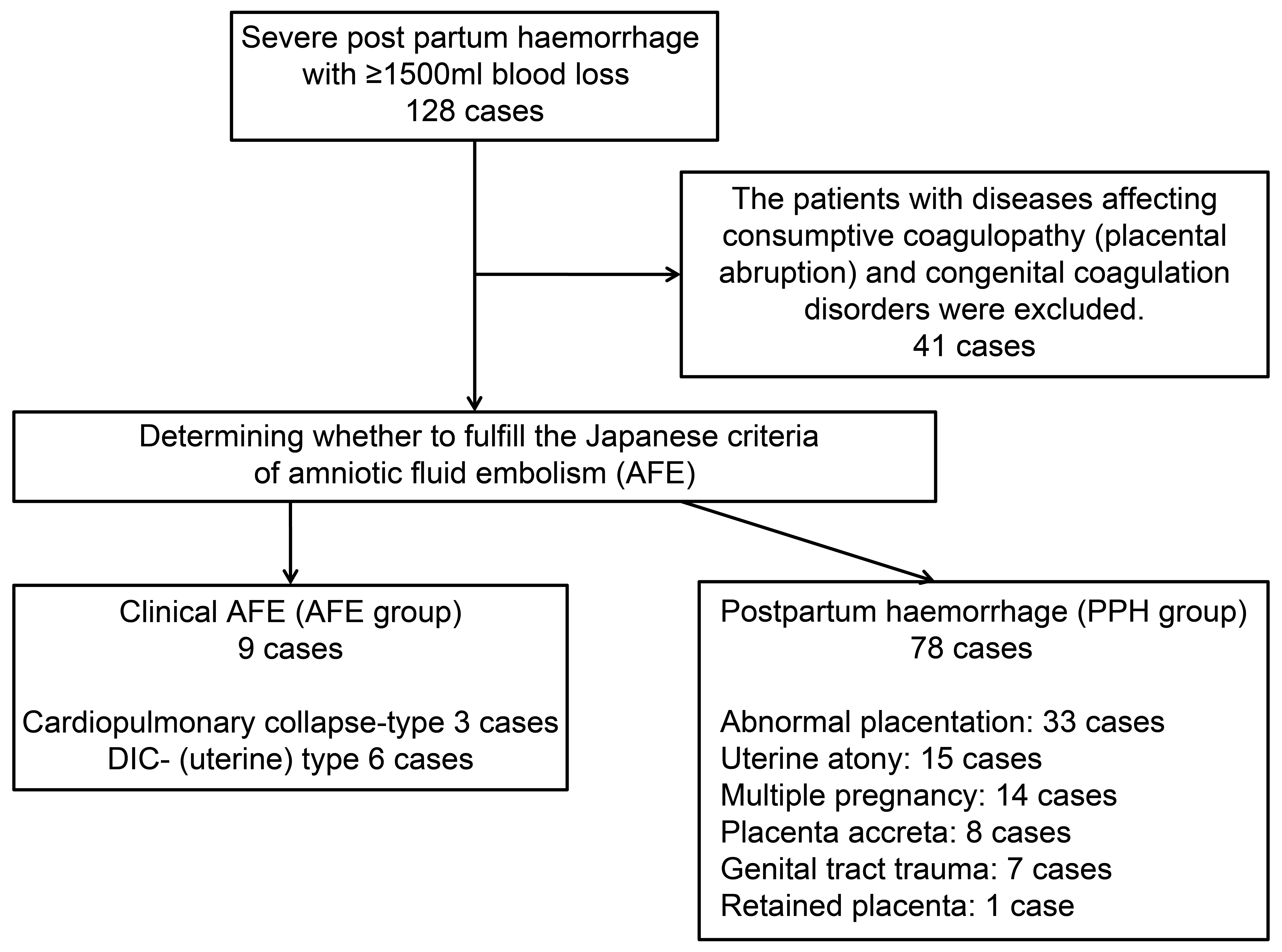
**

**Supplementary Figure S1.** Flow Chart Showing the Patients Enrolled in this Study.

**
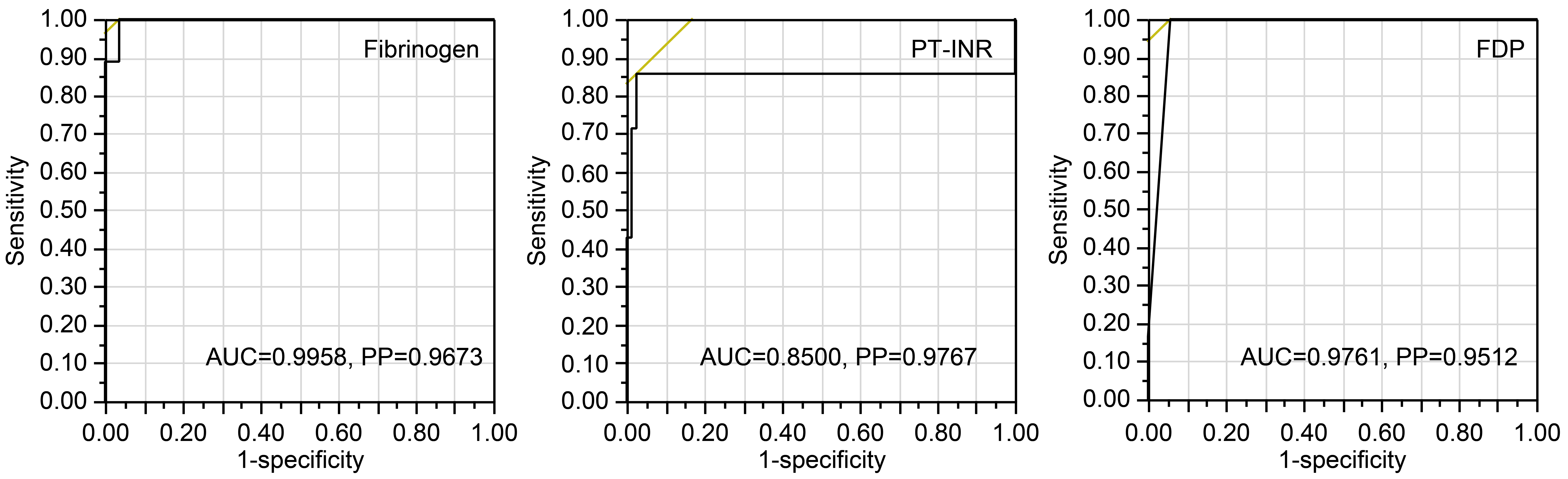
**

**Supplementary Figure S2.** ROC Curves, Cut-off Values, and Predictive Powers of Factors Exhibiting Strong Correlations with AFE.

Cut-off values and predictive powers (PP) for AFE included fibrinogen: cutoff 132 mg/dL, PP=0.9673; PT-INR: cutoff 1.44, PP=0.9767; FDP: cutoff 80.0 μg/dL, PP=0.9512.

**Supplementary Tables**

**Supplementary Table S1.** Japanese Criteria for Amniotic Fluid Embolism (AFE).

| (1) If symptoms appeared during pregnancy or within 12 h of delivery.  (2) If any intensive medical intervention was conducted to treat one or more of the following symptoms/ diseases:  A) Cardiac arrest  B) Severe bleeding of unknown origin within 2 h of delivery (≥1500 mL)  C) Disseminated intravascular coagulation  D) Respiratory failure  (3) If the findings or symptoms obtained cannot be explained by other diseases.  A clinical diagnosis of AFE can be made if the pathological condition meets the above three criteria. Because these diagnostic criteria serve the purpose of making a clinical diagnosis and being able to promptly provide treatment, the pathological conditions that meet them may include diagnoses other than AFE. |
| --- |

**Supplementary Table S2.** Background of the Patients Enrolled, including Association with the Risk Factors of AFE.

|  | AFE group (n=9) | Non-AFE PPH group (n=78) | p-value |
| --- | --- | --- | --- |
| Maternal age | 35.0 ±1.9 | 34.9 ±0.6 | 0.964 |
| Multipara | 4 (44.4%) | 38 (48.7%) | 0.808 |
| Parturient inducer | 4 (44.4%) | 6 (7.7%) | 0.001* |
| Caesarean section | 4 (44.4%) | 66 (84.6%) | 0.004* |
| Operative vaginal delivery | 0 (0%) | 6 (7.7%) | 0.388 |

AFE: amniotic fluid embolism; PPH: postpartum haemorrhage.

**Supplementary Table S3.** Vital Signs and Blood Loss at Onset for Each Group.

|  | AFE group (n=9) | | non-AFE PPH group (n=78) | | p-value |
| --- | --- | --- | --- | --- | --- |
|  | Median (range) | Mean ± SD | Median (range) | Mean ± SD |  |
| Systolic blood pressure (mmHg) | 87 (62 - 120) | 87.7 ±15.9 | 96 (50 - 136) | 96.2 ±16.8 | 0.179 |
| Heart rate (/min) | 97 (73 – 130) | 97.7±21.1 | 95 (60 – 139) | 95.9 ±19.2 | 0.831 |
| Shock index | 1.1 (0.8 – 2.1) | 1.2 ±0.47 | 1.0 (0.6 – 2.5) | 1.0 ±0.37 | 0.337 |
| Blood loss at onset (mL) | 1506 (1-2719) | 1426 ±1160.3 | 1843 (1300-5000) | 2030 ±633.9 | 0.0163* |
| Blood loss at two hours after onset (mL) | 3304 (500-17860) | 5171 ±5339.9 | 1996 (1500-6470) | 2320 ±979.1 | <0.0001* |

AFE: amniotic fluid embolism; PPH: postpartum haemorrhage.
